# ALKAPTONURIA ASSOCIATED WITH DEGENERATIVE COLLAGENOUS PALMAR PLAQUES

**DOI:** 10.4103/0019-5154.55650

**Published:** 2009

**Authors:** Biju Vasudevan, M P S Sawhney, S Radhakrishnan

**Affiliations:** *From the Department of Dermatology, Base Hospital, Delhi Cantt, New Delhi - 110 010, India. E-mail: biju.deepa@rediffmail.com*

Sir,

Alkaptonuria is a rare inherited genetic disorder of tyrosine metabolism caused by a defect in the enzyme homogentisic acid oxidase. The clinical manifestations are caused by the accumulation of homogentisic acid (HGA) in the fibrillary collagens of connective tissues to which it binds irreversibly.[1] Ochronosis is the most common and earliest clinical manifestation to appear. Elevation in urinary HGA excretion by a factor of 100-600 or the detection of HGA in plasma is considered diagnostic for alkaptonuria.[2] Herein, we present a case of Alkaptonuria with an unusual cutaneous finding not described earlier in literature.

A 60-year-old lady, resident of Shimla and farmer by profession presented to the Skin OPD with complaints of pigmentation of the face and hands of 15 years duration. She had initially noticed dark color on her cheeks, which spread to the forehead and later involved the hands. She also had complaints of low backache and pain in the knee and hip joints of the same duration. She noticed that her urine was dark in color since the past two years. Similar complaints were present in her elder sister. There was no history suggestive of any autoimmune disease.

**Figure 1:**
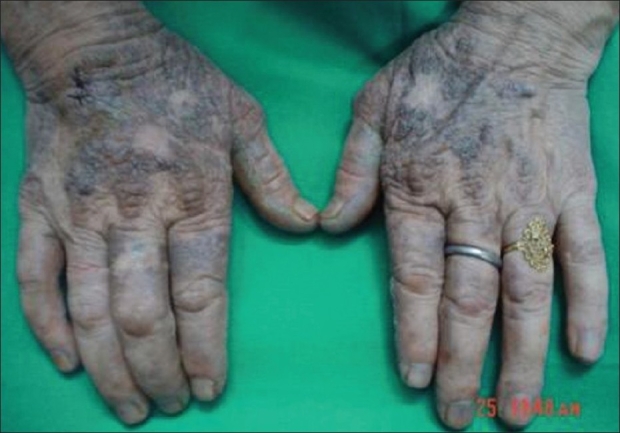
Pigment granules on dorsum of hands

**Figure 2:**
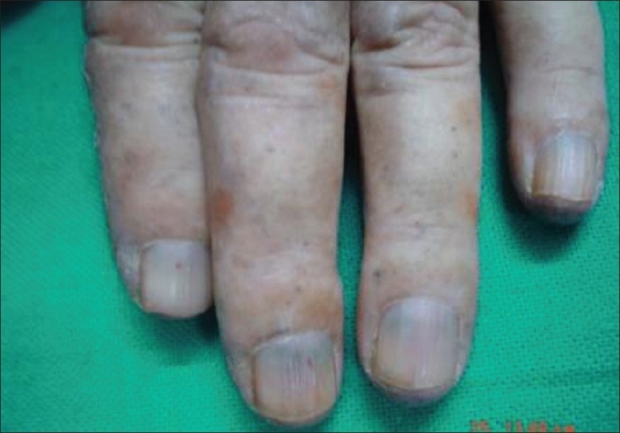
Bluish discoloration of nails

**Figure 3:**
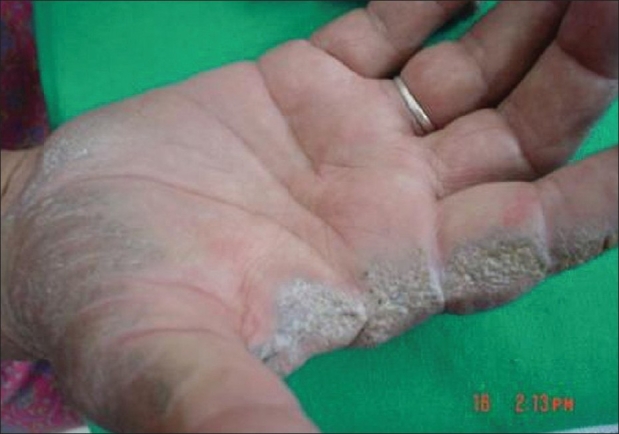
Multiple collagenous plaques on palms

Examination showed normal vital and systemic parameters. There was severe restriction associated with painful movements in both hip and knee joints. Dermatological examination revealed hyperpigmentation of the face in a butterfly pattern including the tip of the nose. Dorsum of the hand and digits showed translucent grouped papules appearing like they were filled with dark granules [Figure 1]. Hyperpigmentation was also present on the dorsum of both feet. There was bluish pigmentation of the nail beds of all fingers and toes [Figure 2]. Both sclerae showed blackish discoloration. The helixes of both ears were rigid and similarly pigmented. In addition to the above dermatological findings, there was an interesting finding that the borders and palmar aspect of the hand showed multiple crateriform plaques [Figure 3]. These plaques were well defined, firm, nontender, and nonscaly.

**Figure 4:**
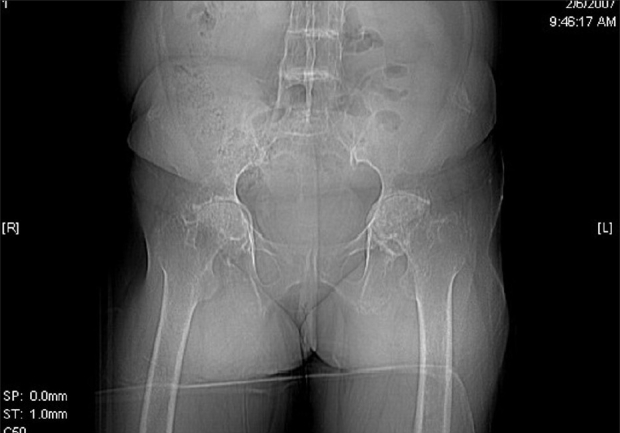
Ochronotic arthropathy of hip joints

**Figure 5:**
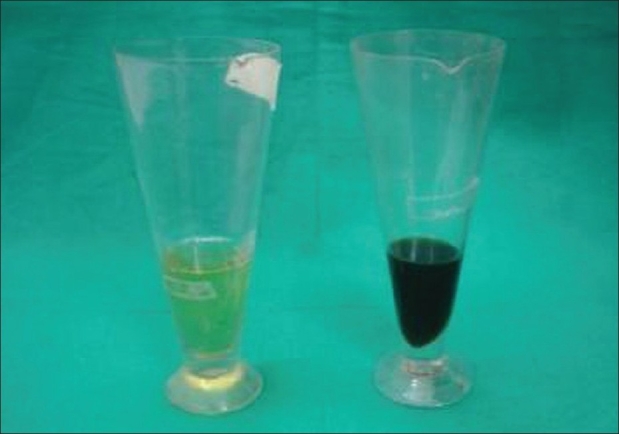
Urine examination showing progressive homogentisic aciduria

Investigations showed a normal blood count while liver and kidney function tests were within normal limits. ANA was negative. Skin biopsy from the crateriform plaques on the palms showed a hyperkeratotic epidermis with intermittent thin or absent stratum corneum. Pigment granules were found in large amounts below the corneal layer. A dense lymphocytic infiltrate with degenerated collagen fibres were present in the dermis. The X-ray of both hips and knees showed changes consistent with osteoarthritis. The X-ray of lumbo-sacral spine revealed ankylosis while MRI showed disc degeneration of the entire lumbar spine and herniation between L4-L5 and L5-S1. CT scan of the spine showed intervertebral disc calcification while that of the sacro-iliac joints and hips showed irregularity of articular margins, reduction in joint space and floating osteophytes [Figure 4]. Urine turned progressively darker with time on naked eye examination [Figure 5]. Ferric chloride and silver nitrate tests done on urine were positive. Urine spectrophotometry was positive for homogentisic acid. The patient was advised to take a low-protein diet and Tab Vit C 1gram/day. She has also undergone both hip and knee replacements with good improvement in mobility.

Diagnosis in the present case was made by the presence of the classical triad of Alkaptonuria, namely ochronosis, dark urine and arthropathy. However in addition she had multiple crateriform plaques on the palms, which has never been reported in literature earlier.

Associations reported with Alkaptonuria so far only include anterior megalophthalmos and lamellar cataract.[3] Recently, a case of acrokeratoelastoidosis has been reported in association with Alkaptonuria.[4] Acrokeratoelastoidosis is a rare clinical entity that presents with hyperkeratotic lesions on the margins of the hands and feet. Costa has described 13 cases of acral keratotic lesions, few of which resemble the above-mentioned crateriform plaques, but none had any associations with Alkaptonuria or any other disease conditions.[5] Several other conditions can have keratotic crateriform papules coalescing to form plaques usually along the borders of hands and feet including mosaic acral hyperkeratosis, verruca plana, acrokeratosis verruciformis of Hopf, xanthoma, keratoelastoidosis marginalis, and digital papular calcinosis.[6] However, our patient had only palmar lesions with sparing of feet.

Degenerative collagenous plaques of the hands is a disorder characterized by linear or bandlike coalesced crateriform papules on the sides of hands or fingers due to degeneration of collagen fibres.[7] Our case probably has a variant of this disorder. This case has been presented for the unique association of degenerative collagenous plaques on palms with Alkaptonuria, which by itself is a rare entity in India.
